# Hydrogen Embrittlement Behavior and Applicability of X52 Steel in Pure Hydrogen Pipelines

**DOI:** 10.3390/ma18143417

**Published:** 2025-07-21

**Authors:** Tianlei Li, Honglin Zhang, Wentao Hu, Ke Li, Yaxi Wang, Yuanhua Lin

**Affiliations:** 1 State Key Laboratory of Oil and Gas Reservoir Geology and Engineering, Southwest Petroleum University, Chengdu 610500, China; litianlei_sw@cnpc.com.cn (T.L.); hwt2481559892@163.com (W.H.); 2 School of New Energy and Materials, Southwest Petroleum University, Chengdu 610500, China; 3 Southwest Branch Co., China Petroleum Engineering Construction Corp., Beijing 100120, China; like1_sw@cnpc.com.cn (K.L.); wangyaxi_sw@cnpc.com.cn (Y.W.)

**Keywords:** X52 steel, hydrogen embrittlement, fatigue crack growth, tensile properties, fracture toughness

## Abstract

This study investigates the mechanical behavior of X52 steel pipes and their weld regions under pure hydrogen transport conditions, with a focus on assessing potential hydrogen embrittlement risks. Through experimental analysis, the research evaluates how different pipeline regions—including the base metal, weld metal, and heat-affected zones—respond to varying hydrogen pressures. Key mechanical properties such as elongation, fracture toughness, and crack growth resistance are analyzed to determine their implications for structural integrity and safety. Based on the findings, this study proposes criteria for the safety evaluation of X52 pipelines operating in hydrogen service environments. The results are intended to inform decisions regarding the repurposing of existing pipelines or the design of new infrastructure dedicated to pure hydrogen transport, offering insights into material performance and critical safety considerations for hydrogen pipeline applications.

## 1. Introduction

In recent years, hydrogen has emerged as a pivotal element in the global transition to sustainable and clean energy systems, driven by the urgent need to mitigate environmental challenges and reduce carbon emissions [1]. Given its potential to decarbonize industries and serve as a renewable energy carrier, hydrogen is increasingly being integrated into various energy systems [2,3], including natural gas pipelines. However, hydrogen’s unique characteristics, such as its low molecular weight and high diffusivity, pose significant challenges for its storage, transportation, and safe utilization, particularly within existing pipeline infrastructure [4]. Ensuring the safe and efficient transportation of hydrogen through pipelines is critical for realizing its role as a cornerstone of future energy systems [5,6].

Hydrogen embrittlement, a process where hydrogen alters the mechanical properties of materials, represents one of the most critical challenges in utilizing hydrogen in pipeline systems [7,8,9]. This phenomenon occurs when hydrogen atoms diffuse into metallic materials, reducing their ductility and toughness, and ultimately leading to cracking and failure [10,11]. High-strength steels, such as X52 pipeline steel commonly used in natural gas transmission pipelines, are particularly susceptible to this issue [12]. Hydrogen exposure can significantly degrade the mechanical properties of X52 steel, affecting its tensile strength, fracture toughness, and fatigue resistance [13,14,15]. Consequently, understanding the impact of hydrogen on pipeline materials is essential to ensure the long-term integrity and safety of hydrogen-transmitting infrastructure [16].

Previous studies have examined the behavior of pipeline steels under hydrogen exposure, with particular emphasis on hydrogen-induced cracking (HIC) and hydrogen-induced stress cracking (HISC), which can result in catastrophic failures in pipeline systems [17,18]. Research indicates that the extent of hydrogen embrittlement is influenced by factors such as material strength, hydrogen pressure, and environmental conditions. For X52 steel, exposure to hydrogen environments leads to notable changes in tensile properties, including reduced plasticity and increased susceptibility to stress-induced fractures. Additionally, hydrogen-induced fatigue crack growth accelerates, diminishing the material’s fatigue life, which is crucial for ensuring the safe operation of pipelines over extended periods [19,20,21].

This study investigates the mechanical degradation of X52 pipeline steel in hydrogen-rich environments, focusing on hydrogen-induced cracking, tensile property reduction, and fatigue crack growth behavior. Through experimental testing under varying hydrogen pressures, the research evaluates key failure mechanisms to assess the material’s suitability for hydrogen transportation. The study aims to establish a comprehensive understanding of X52 steel’s performance in hydrogen service, providing critical data for pipeline safety and reliability. Additionally, the findings contribute to the development of enhanced materials and protective technologies to mitigate hydrogen embrittlement risks. By addressing these challenges, the research supports the broader adoption of hydrogen as a sustainable energy carrier while ensuring pipeline integrity.

## 2. Main Failure Behaviors of Metallic Materials in Hydrogen Environments

### 2.1. Hydrogen-Induced Cracking

Hydrogen-induced stress cracking (HISC) is characterized by its delayed nature, requiring continuous exposure to both hydrogen and stress for a brittle fracture to occur. In most cases, the stress leading to a fracture is lower than the material’s yield strength, posing a risk to the safe operation of pipelines. HISC is most likely to occur at room temperature, and in hydrogen-containing environments, the fracture toughness of sensitive materials is significantly lower than in inert gas atmospheres [22,23]. The degree of performance loss is influenced by both the material and environmental factors. The critical cracking stress intensity factor (*K_IH_*), which indicates hydrogen-induced fracture toughness, is a function of the strength level and hydrogen pressure environment. When the environmental stress intensity factor is below *K_IH_*, cracking will not occur. Therefore, by assessing the stress value and defect size at pipeline defects, the cracking risk of the pipeline can be evaluated. Blunt defects, such as corrosion, may not generate significant stress, whereas sharp defects, such as cracks, can result in severe stress concentration and should be a major focus [24,25].

### 2.2. Reduction in Tensile Properties

High-grade pipeline steels exhibit a reduction in tensile plasticity and strength under the influence of hydrogen, especially in the presence of defects, where the reduction is more pronounced. A premature rupture of tightened samples is also an indication of strength degradation. Therefore, changes in the tensile properties of pipeline steel materials in hydrogen environments are crucial for evaluating the hydrogen embrittlement sensitivity of pipeline steels.

Boukortt et al. [26] focused on the impact of hydrogen on the mechanical properties of X52 pipeline steel. They found that the presence of hydrogen caused a 2.5% decrease in the yield strength, and the elongation after a fracture decreased significantly by 38%. On the other hand, the studies of Wang Rong and Zhao Ying et al. [27,28,29] showed that after electrochemical hydrogen charging and when performing slow tensile tests on X70 pipeline steel, the strength remained almost unchanged, but the plasticity and fracture toughness decreased as the hydrogen-charging current density increased. However, this decrease tended to level off after reaching a certain threshold. Additionally, Zhou [30] compared the tensile properties of smooth and notched samples in hydrogen environments, finding that the mechanical property degradation of notched samples was more severe than that of smooth samples under the same hydrogen atmosphere.

### 2.3. Hydrogen-Induced Fatigue Crack Growth

In metals, the initial formation of fatigue cracks often occurs in specific regions, such as the persistent slip bands [31], grain boundaries, and inclusion sites. The research team of Briottet [32] revealed the significant effect of high-pressure hydrogen gas on crack initiation in CrMo steel: as the hydrogen pressure increased from 0.5 MPa to 30 MPa, the number of cycles required for crack initiation decreased dramatically to one-tenth. On the other hand, Lee et al. [33] focused on the effects of hydrogen on fatigue crack behavior in low-carbon steel at extremely low growth rates. They found that cracks remained stationary in nitrogen environments but began to propagate near the crack tip under low-pressure hydrogen conditions.

An et al. [34] conducted low-cycle fatigue and crack growth tests to explore the specific effects of different hydrogen partial pressure conditions on the hydrogen embrittlement sensitivity of X80 steel in a hydrogen-containing environment with a total pressure of 12 MPa. The study showed that as the hydrogen partial pressure increased, the fatigue cycle life of notched X80 samples decreased significantly, while the crack growth rate of compact tensile specimens increased sharply. Compared to the nitrogen environment, under a hydrogen partial pressure of 0.2 MPa, the fatigue failure cycle life of X80 steel decreased by 20%, and the fatigue crack growth rate increased by a factor of 7. When the hydrogen partial pressure further increased to 8 MPa, the reduction in the failure cycle life reached 90%, and the crack growth rate doubled. An further emphasized that with increasing hydrogen pressure, the increased crack growth rate was the primary factor contributing to the shortened fatigue life of X80 steel.

Meng et al. [35] studied the effects of a natural gas/hydrogen gas mixture containing 0% to 50% (by volume) hydrogen on the mechanical properties of X80 pipeline steel under a pressure of 12 MPa. The results showed that as the hydrogen content increased, the hydrogen embrittlement sensitivity of X80 steel also increased, and the fatigue crack growth rate accelerated significantly.

Similarly, in recent studies, Nguyen et al. [36] found that in the pressure range of 5 to 10 MPa, the hydrogen embrittlement sensitivity of X70 steel increased with the increase in hydrogen content. Particularly, when the hydrogen volume fraction reached 0.7%, the fracture mode of X70 steel underwent a significant shift from a ductile fracture to a brittle fracture.

The fatigue crack growth rate is one of the key indicators for evaluating the hydrogen embrittlement sensitivity of materials [37,38,39]. Additionally, the microstructure of pipeline steels, especially the grain size of ferrite, plays an important role in the hydrogen-assisted crack growth effect. The ferrite grain size not only controls the strength of the pipeline steel, thereby influencing its hydrogen embrittlement sensitivity, but also determines the diffusion behavior of hydrogen atoms within the material. Specifically, refining ferrite grains can enhance the strength of pipeline steels but may also exacerbate hydrogen embrittlement, as the increased number of grain boundaries provides more pathways for hydrogen atom diffusion [40].

### 2.4. Fracture Toughness Reduction

**Figure 1:**
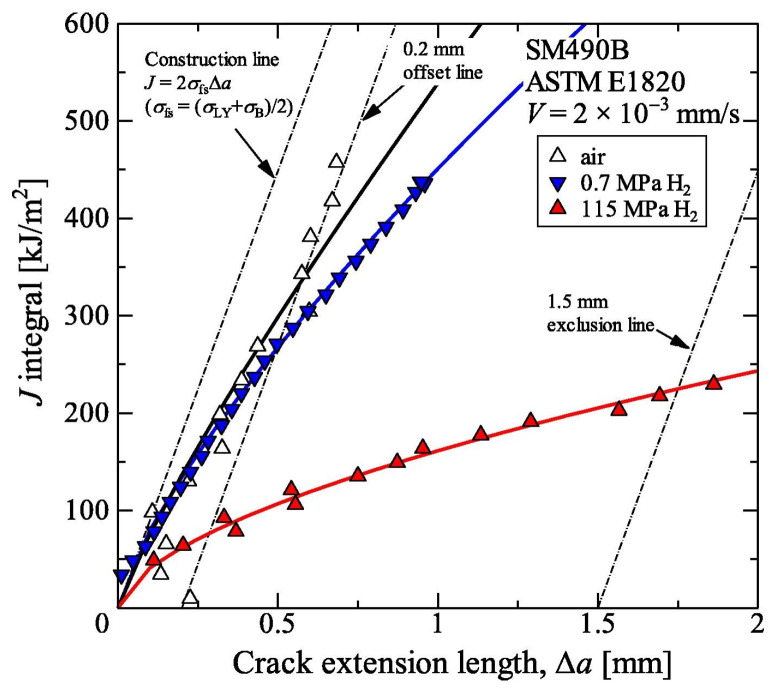
J-integral resistance curves of JIS-SIM490B in nitrogen and hydrogen environments [52].

For low-strength carbon steels, subcritical crack growth is not observed under static loading; however, as the load increases, the presence of hydrogen significantly affects the material’s fracture toughness [41]. Pipeline steels, being materials with high toughness, typically evaluate fracture toughness using the elastic–plastic J-integral method. As shown in Figure 1, fracture toughness tests were conducted on SM490B steel under different hydrogen pressures (low and high pressure), and the results indicated that low-pressure hydrogen had little impact on fracture toughness. In contrast, as the hydrogen pressure increased, a clear downward trend in fracture toughness was observed.

Fracture toughness is the intrinsic ability of a material to resist an unstable fracture under an applied load, and it is a characteristic independent of the specimen, crack shape, and magnitude of the applied stress. In engineering, fracture toughness has become a key parameter for assessing the mechanical performance of materials, as it reflects a material’s behavior under specific environmental conditions. The latest international research has provided detailed procedures for the testing of fracture toughness and established corresponding national standards such as ASTM E1820-11 [42] and ASTM E1290-11 [43]. Although a large amount of research has been conducted on the fracture toughness of pipeline steel in a hydrogen environment, the conclusions remain controversial.

Chatzidouros et al. [44,45,46] investigated the effect of electrochemical hydrogen charging on the fracture toughness of X52, X65, and X70 pipeline steels in three-point bending specimens. Their findings showed that as the hydrogen-charging current density increased, the fracture toughness of all three steels decreased, with the rate of decrease gradually slowing down. Among them, the fracture toughness of X65 decreased most significantly, which contradicts the previously held view that hydrogen embrittlement sensitivity increases with higher steel grades. The authors speculated that this may be due to the martensite–austenite phase in X65 steel, which is more susceptible to hydrogen damage, whereas the high dislocation density of low-carbon bainite acts as a hydrogen trap, leading to a significant reduction in the fracture toughness in a hydrogen environment.

Zhang et al. [47,48] tested the fracture toughness and fatigue life of X80 pipeline steel in hydrogen environments and found that the presence of hydrogen resulted in a decrease in both the fracture toughness and fatigue life. The fracture surface morphology showed fewer ductile dimples, more microcracks, and a transition from a ductile fracture to a brittle quasi-cleavage fracture. Briottet et al. [49] used a multi-sample approach to study the fracture toughness of X80 pipeline steel in a 300-bar hydrogen environment. They found that high-pressure pure hydrogen significantly reduced the fracture toughness of X80 steel. Additionally, first-principle calculations and linear elastic fracture mechanics models have been used to simulate the adsorption behavior of hydrogen atoms on the surface of pipeline steels and their effect on fracture toughness [50,51].

## 3. Experimental Materials and Testing Methods

### 3.1. Materials

The material selected for this experiment is X52 steel. X52 steel belongs to pipeline steel, and its characteristics and applications are as follows.

High strength: The yield strength is approximately 360 megapascals, and the tensile strength is no less than 460 megapascals, capable of withstanding considerable pressure and load.

Good toughness: It has good impact resistance even in low-temperature environments and is suitable for use in complex working conditions.

Excellent weldability: It can be connected through conventional welding processes, and the performance of the welded joint is stable.

Strong corrosion resistance: It has a certain ability to resist corrosion to the conveyed medium (such as natural gas, petroleum, etc.) and the external environment.

It is mainly used for manufacturing pipelines for transporting natural gas, oil, and other media, and it is suitable for long-distance gas transmission pipelines, oil transportation pipelines, and other projects.

### 3.2. Experimental Tests

The objective of this study is to evaluate the hydrogen embrittlement behavior of X52 steel and its applicability in pure hydrogen pipelines. To achieve this, multiple testing methods were designed to systematically assess the mechanical performance of X52 steel in different environmental conditions. These tests cover several aspects, including metallographic analysis, hardness testing, tensile performance, fracture toughness, and fatigue crack growth. The goal is to analyze the changes in material properties under the influence of hydrogen and provide theoretical support for the application of X52 steel.

The X52 steel was provided by Julong Steel Pipe Co., Ltd., Nanjing, China with specifications of φ610 mm × 14.3 mm and φ1219 mm × 27.5 mm. The specific chemical composition and tensile properties of the steel are shown in Table 1 and Table 2.

**Figure 2:**
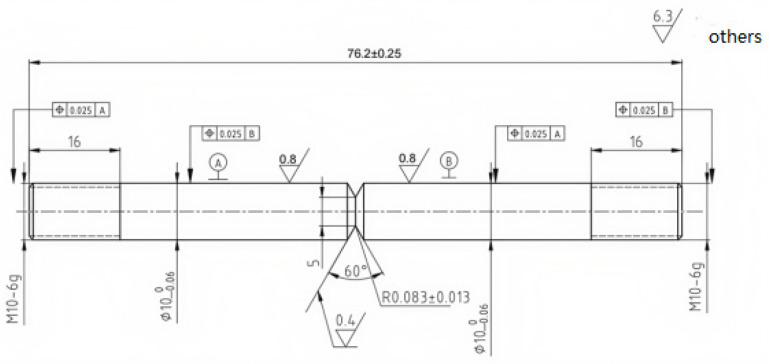
Schematic diagram of tensile specimen with notch.

**Figure 3:**
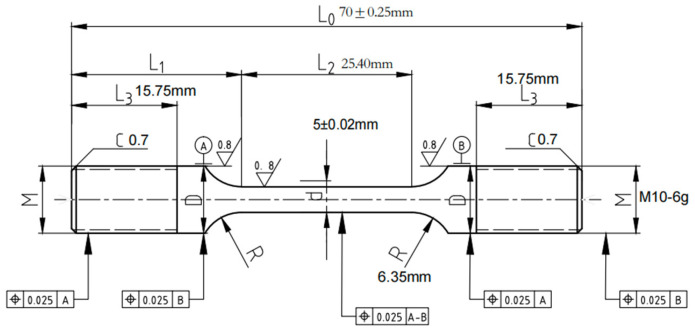
Schematic diagram of smooth tensile specimen.

**Figure 4:**
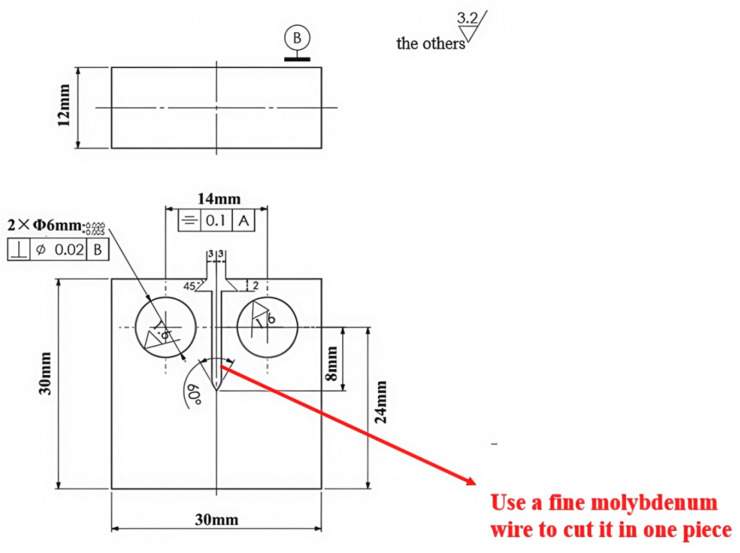
Schematic diagram of compact tension specimen (CT specimen).

**Figure 5:**
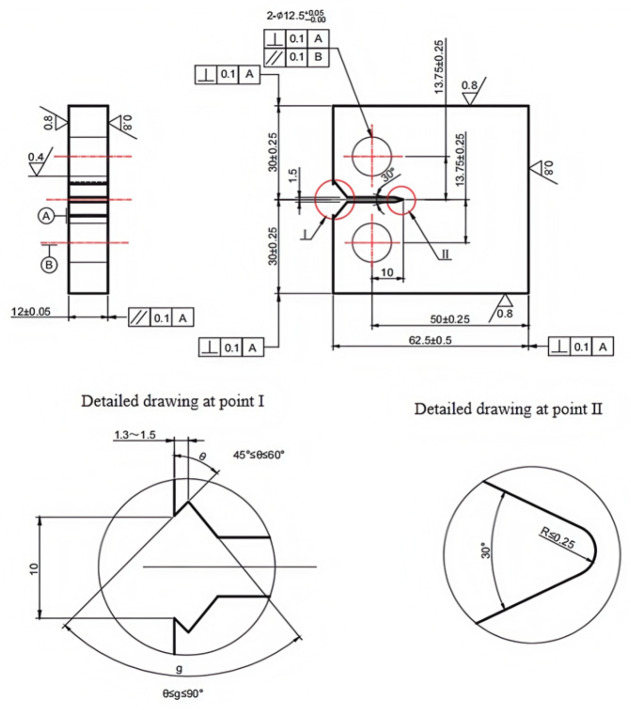
Schematic diagram of fatigue crack growth specimen.

Each test is carried out in strict compliance with relevant international standards and industry specifications, ensuring the reliability and comparability of the results. By employing these methods, this study offers a comprehensive comparison of the mechanical properties of X52 steel under both ambient conditions and hydrogen-containing environments, thereby facilitating an in-depth analysis of the effects of hydrogen embrittlement on pipeline steel performance. The tensile rate of the sample in Figure 2 was set at 5 × 10^−5^ mm/s, that of the sample in Figure 3 was set at 3.5 × 10^−4^ mm/s, the sample in Figure 4 was pre-cracked at 3 mm, the tensile rate was set at 0.012 mm/min, and the load ratio R of the specimen in Figure 5 was 0.1, with sinusoidal loading conducted at a frequency of 1 Hz.

Table 3 summarizes the main testing methods utilized in this study, along with their corresponding standards, equipment, and procedures, which form the experimental basis for subsequent data analysis and discussion.

**Table 3:** Overview of testing methods for evaluating hydrogen embrittlement behavior of X52 steel in pure hydrogen pipelines.

Test Item	Test Method Overview	Relevant Standards/References	Key Testing Content	Instruments/Equipment
Metallographic Analysis	The sample is encapsulated with bakelite and sequentially polished using sandpaper. It is then etched with 4% nitric acid alcohol solution and observed under a metallurgical microscope.	GB/T 13298-2009 [53] (Metallic Microstructure Analysis Methods)	Observation of material microstructure to analyze the metallographic features.	Metallographic Microscope (Shanghai Siyao Optical Instruments Co., Ltd., Shanghai, China), Sandpaper (Sichuan Fengfeng Abrasives Co., Ltd. in Chengdu, China), Polishing Machine (Yongkang Benyuan Industry and Trade Co., Ltd., Yongkang City, China), Etching Solution (Shenzhen Baoxin Chemical Technology Co., Ltd., Shenzhen, China)
Hardness Test	The sample is polished using diamond abrasives on sandpaper, followed by hardness measurements using a microhardness tester.	GB/T 4340.1-2009 [54] (Microhardness Testing Method)	Measurement of the microhardness of the material to evaluate its hardness performance.	DHV-1000Z Microhardness Tester (Changzhou Sanfeng Instrument Technology Co., Ltd., Changzhou, China), Diamond Abrasive Solution (Henan Taizheng New Materials Co., Ltd., in Xinxiang, China)
Tensile Performance Test	Tensile specimens are subjected to testing in both ambient and hydrogen-containing environments, assessing the strength, plasticity, and fracture morphology of the material.	GB/T 228.1-2010 [55] (Tensile Test Methods)	Measurement of tensile strength, elongation, and reduction in area to assess the mechanical performance of the material.	CMT4204 Microcomputer-Controlled Electronic Universal Testing Machine (Mattel Industrial Systems (China) Co., Ltd., Shanghai, China)
Notched Specimen Tensile Test	Notched tensile tests are conducted based on ASTM G 142-98, analyzing the influence of hydrogen on the material’s tensile properties.	ASTM G 142-98 [56] (Notched Tensile Test Methods)	Evaluation of the tensile properties and fracture morphology of materials in hydrogen environments.	CMT4204 Microcomputer-Controlled Electronic Universal Testing Machine (Mattel Industrial Systems (China) Co., Ltd., Shanghai, China)
Smooth Specimen Tensile Test	Slow-strain-rate tensile tests are performed following ASTM G 142-98 [56] and GB/T 15970.7-2017 [57] standards, analyzing the influence of hydrogen on smooth specimens.	ASTM G 142-98 (Tensile Test Methods)	Measurement of the material’s mechanical performance under hydrogen exposure.	Tensile Testing Machine (Jinan Chenda Testing Machine Manufacturing Co., Ltd., Jinan, China), Smooth Specimens (Jinan Chenda Testing Machine Manufacturing Co., Ltd., Jinan, China)
Fracture Toughness Test	CTOD tests are conducted to assess crack tip opening displacement and fracture growth in both ambient and hydrogen-containing environments.	GB/T 2358-2007 [58] (Fracture Toughness Testing Methods)	Evaluation of fracture toughness and crack growth behavior under hydrogen exposure.	CTOD Testing Equipment (Jinan Hengleike Xingke Instrument Co., Ltd., Jinan, China), Specimen (Jinan Hengleike Xingke Instrument Co., Ltd., Jinan, China)
Fatigue Crack Growth Test	Fatigue crack growth tests are conducted following GB/T 6398-2000 [59], with specimens exposed to both ambient and hydrogen-containing environments.	GB/T 6398-2000 (Fatigue Crack Growth Testing Methods)	Measurement of the fatigue crack growth rate and threshold values of materials in hydrogen environments.	Fatigue Testing Machine (China Machinery Testing Equipment Co., Ltd., Changchun, China), C(T) Specimen (China Machinery Testing Equipment Co., Ltd., Changchun, China)

## 4. Experimental Results and Discussion

### 4.1. Metallographic Analysis

**Figure 6:**
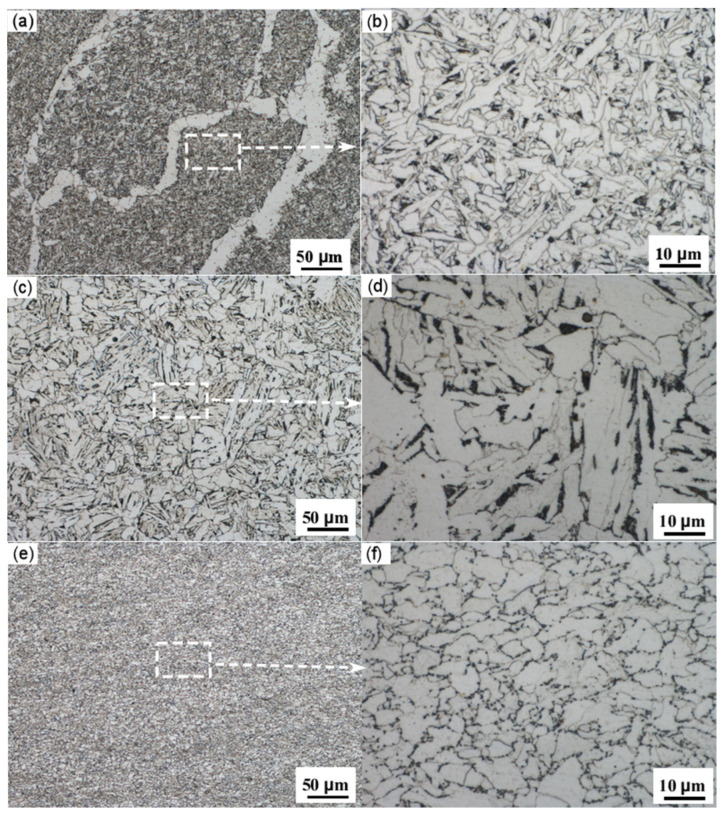
(**a**) Center of the girth weld; (**b**) enlarged view of the centerline of the girth weld; (**c**) HAZ of the girth weld; (**d**) enlarged view of the girth weld HAZ; (**e**) base metal; (**f**) enlarged view of the base metal.

Figure 6 presents metallographic images at different magnifications of the base metal, the center of the straight weld, and the heat-affected zone (HAZ) of the straight weld. It is clearly observed from the images that there are significant differences in the microstructure and grain size between the different regions, and the influence of the pipeline forming process results in relatively poor microstructural uniformity.

The results from the analysis show that the base metal is primarily composed of ferrite and pearlite, with a relatively low content of pearlite [60]. The microstructure of the heat-affected zone of the straight weld mainly consists of ferrite and pearlite, with pearlite appearing as a distinct banded structure. The grain size in this zone is larger. In contrast, the weld metal is predominantly characterized by acicular ferrite, with a relatively uniform grain size and distribution.

**Figure 7:**
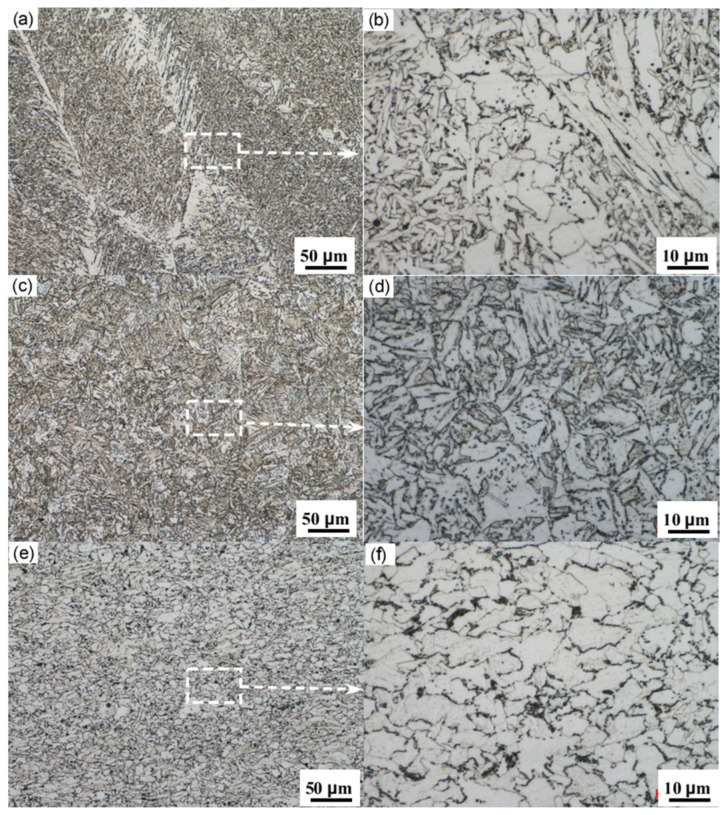
(**a**) Center of the girth weld; (**b**) enlarged view of the centerline of the girth weld; (**c**) HAZ of the girth weld; (**d**) enlarged view of the girth weld HAZ; (**e**) base metal; (**f**) enlarged view of the base metal.

Figure 7 shows the metallographic microstructure of the X52 steel pipe’s girth weld. From the image, it can be observed that both the base metal and the HAZ of the girth weld consist mainly of ferrite and pearlite, with a relatively low content of pearlite and a slight banded distribution. The microstructure of the weld zone exhibits coarse grains and an uneven distribution, displaying characteristics of a Widmanstätten structure. Due to the heat input during welding, the austenite grains have grown significantly. Under rapid cooling conditions, many parallel ferrite needles form within the coarse austenite grains, and the remaining austenite between the ferrite needles ultimately transforms into pearlite. This microstructure typically exhibits poor overall mechanical performance.

### 4.2. Hardness Analysis

The hardness values of the X52 steel pipeline in the straight weld and girth weld are shown in Table 4. The data indicate that the hardness value at the center of the straight weld is higher than that of the base metal and the heat-affected zone, suggesting that the straight weld region may be more sensitive. In contrast, the hardness values of the base metal and heat-affected zone in the girth weld are higher than those at the center of the girth weld.

**Figure 8:**
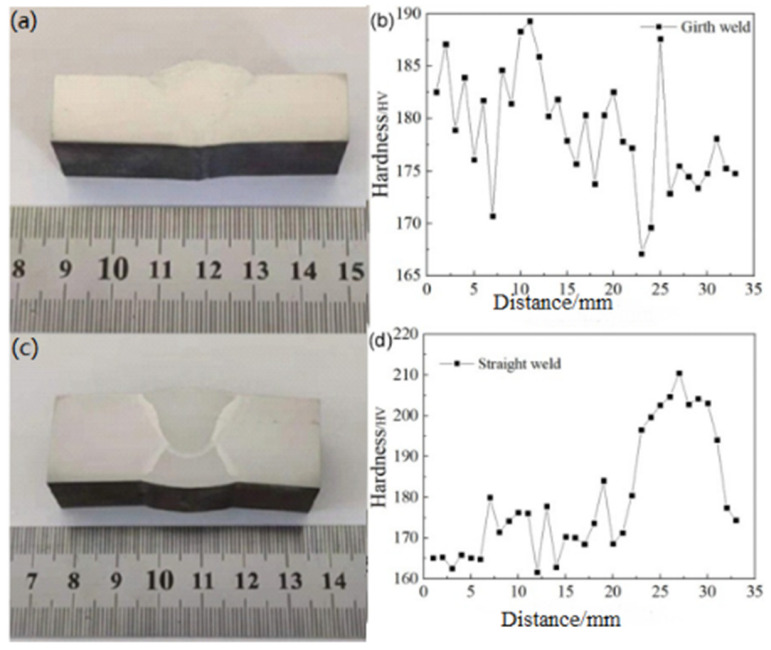
Hardness distribution at the straight weld and girth weld positions of X52 steel pipe: (**a**,**b**) straight weld; (**c**,**d**) girth weld.

To better understand the variation in hardness values from the weld center to the base metal, multiple points were tested within the region. To ensure the accuracy of the experimental data, the indentations were spaced 2–3 times the diagonal length of the indentations apart. The hardness distribution is shown in Figure 8. As observed in Figure 8, there is a sharp increase in hardness at the transition between the heat-affected zone and the weld, and the material’s inhomogeneity at this location contributes to an increased sensitivity. Therefore, both the weld and heat-affected zone require particular attention (Table 5).

### 4.3. Tensile Property Testing

According to GB/T 9711-2017 “Steel Pipes for Petroleum and Natural Gas Industry—Pipeline Transport Systems,” mechanical property tests were conducted on the X52 steel base metal, straight weld, and girth weld. The results are shown in Table 4. As seen from the table, the yield strength at the weld center is almost identical to that of the base metal. The tensile strength of the X52 steel girth weld is higher, but the elongation of both the straight and girth welds is lower than that of the base metal.

### 4.4. Notch Tensile Test

**Figure 9:**
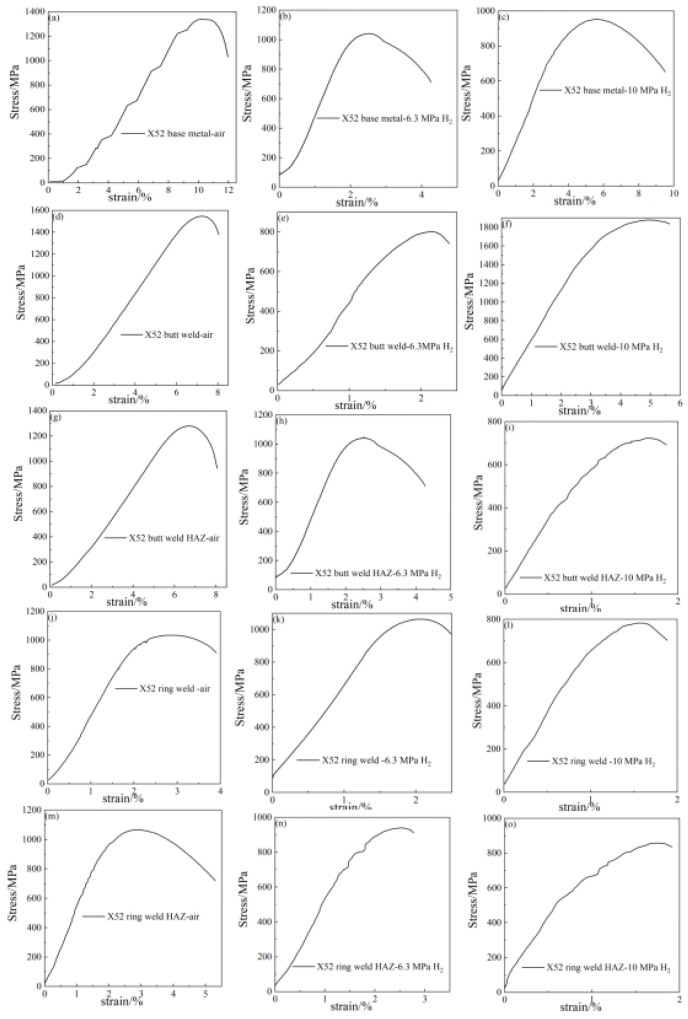
Notch tensile curves of X52 steel pipe: base metal: (**a**–**c**); straight weld: (**d**–**f**); straight weld HAZ: (**g**–**i**); girth weld: (**j**–**l**); girth weld HAZ: (**m**–**o**).

The notch tensile curves and fracture morphology of X52 steel pipes at different sampling positions under air, 6.3 MPa hydrogen, and 10 MPa hydrogen conditions are shown in Figure 9. From the tensile curves, it can be seen that, compared to the base metal, the notch tensile strength of the X52 steel base metal, girth weld, and girth weld heat-affected zone changes little in the 6.3 MPa hydrogen environment, while the notch tensile strength of the straight weld and straight weld heat-affected zone significantly decreases. This may be due to the presence of defects and stress concentration in the tensile specimens, resulting in lower tensile strength. Under the 10 MPa hydrogen condition, the notch tensile strength at all positions decreases, but the change is not significant. The reduction in cross-sectional shrinkage at all positions of the X52 steel pipe under the 6.3 MPa hydrogen condition is higher than that under the 10 MPa hydrogen condition, indicating that the material’s cross-sectional shrinkage significantly decreases with the increase in hydrogen partial pressure.

**Figure 10:**
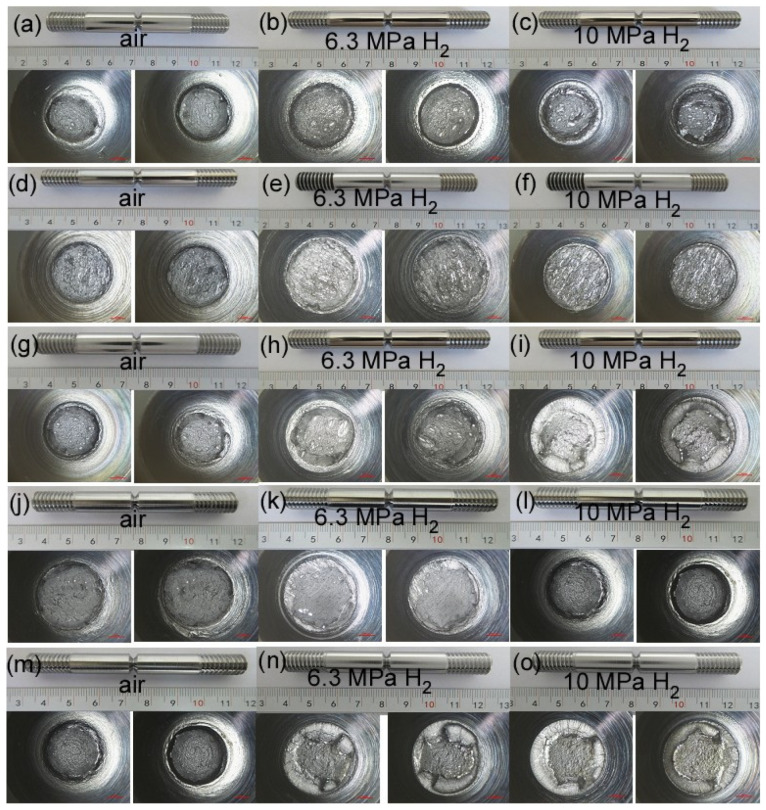
The macro-fracture morphology of the post-tensile specimens: base metal: (**a**–**c**); straight weld: (**d**–**f**); straight weld HAZ: (**g**–**i**); girth weld: (**j**–**l**); girth weld HAZ: (**m**–**o**).

Figure 10 shows the micro-fracture morphology of the post-tensile specimens. From this figure, it is evident that under the 10 MPa hydrogen condition, the fracture edge exhibits an obvious brittle fracture, and a brittle fracture is also observed at the straight weld position and the heat-affected zone of the girth weld under the 6.3 MPa hydrogen condition. This indicates that X52 steel is at a higher risk of hydrogen embrittlement under the 10 MPa hydrogen environment.

**Figure 11:**
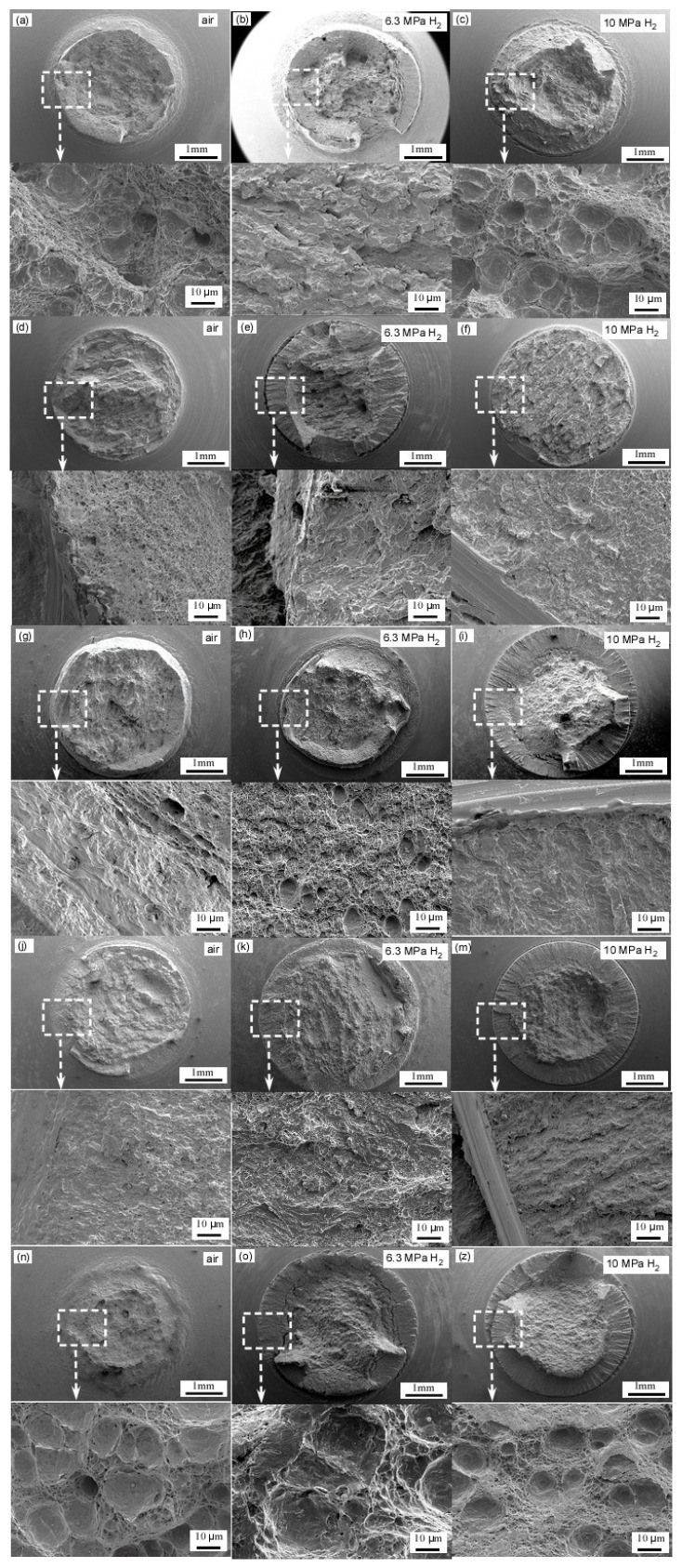
Microstructural fracture in X52 steel pipes across different regions under varying hydrogen concentrations: base metal: (**a**–**c**); straight weld: (**d**–**f**); straight weld HAZ: (**g**–**i**); girth weld: (**j**–**m**); girth weld HAZ: (**n**–**z**). The graph to which each arrow points represents an enlarged view of the corresponding box area.

Figure 11 displays the microstructural fracture in X52 steel pipes across different regions under varying hydrogen concentrations. The fracture surface of the X52 steel base material in an air environment (Figure 11a) is predominantly characterized by a ductile fracture, exhibiting a large number of uniformly distributed dimples. The significant depth and size of these dimples indicate that the material possesses a high plastic deformation capability under hydrogen-free conditions. In a 6.3 MPa hydrogen environment (Figure 11b), the density of dimples on the fracture surface decreases, with shallow dimples or quasi-cleavage surfaces appearing in localized regions (as indicated by arrows in Figure 11b). This suggests that hydrogen introduction reduces local plasticity. Hydrogen atoms tend to accumulate at ferrite–pearlite interfaces, acting as microcrack initiation sites, which aligns with the findings in reference [40] that “the pearlite band serves as a hydrogen diffusion barrier, whereas the ferrite band acts as the preferred diffusion path.” In a 10 MPa hydrogen environment (Figure 11c), the fracture surface exhibits pronounced brittle characteristics, dominated by quasi-cleavage and intergranular fractures, with nearly no observable dimples. The elevated hydrogen pressure enhances hydrogen adsorption at grain boundaries, leading to significant grain boundary weakening. This observation correlates well with the experimental data in Table 6, where the cross-sectional reduction rate markedly decreases (from 0.30 to 0.37) under a 10 MPa hydrogen environment.

The fracture surface of the straight weld zone exhibits a mixed morphology of dimples and cleavage due to welding residual stress and micro-defects such as pores and inclusions (Figure 11d). Under a hydrogen environment at 6.3 MPa, the proportion of cleavage areas significantly increases, with cracks propagating along the weld centerline (as indicated by the arrow in Figure 11e). This phenomenon may be attributed to the high hardness of the weld zone (with a hardness value of 202.8 HV at the center of the straight weld, as shown in Table 4) and its banded structure. At 10 MPa hydrogen pressure, the fracture surface becomes entirely brittle, and the crack propagation path exhibits bifurcation characteristics (Figure 11f), indicating that increased hydrogen pressure further reduces the fracture resistance of the weld. The non-uniformity of the microstructure in the heat-affected zone of the straight weld (Figure 6) results in varying sensitivities to hydrogen embrittlement. Under a 6.3 MPa hydrogen environment, intergranular cracking is observed on the fracture surface (Figure 11h), consistent with the findings in reference [25] that the pearlite–ferrite non-banded structure in the HAZ offers weak resistance to hydrogen diffusion. At 10 MPa hydrogen pressure, the fracture surface displays continuous cleavage steps (Figure 11i), suggesting an accelerated hydrogen-induced crack propagation rate.

The fracture surface of the circumferential weld in an air environment is predominantly characterized by dimples; however, the size of these dimples is smaller than that observed in the base material (Figure 11j). This difference may be attributed to the formation of acicular ferrite during the welding process (Figure 7). In a hydrogen environment, the dimple morphology is progressively replaced by cleavage surfaces. Notably, under a 10 MPa hydrogen pressure, secondary cracks extending along the welding direction emerge on the fracture surface (Figure 11k), which highlights the hydrogen enrichment effect in the high-stress regions of the circumferential weld. The heat-affected zone (HAZ) of the circumferential weld exhibits ductile fracture characteristics similar to those of the base material in an air environment (Figure 11m). However, under a 6.3 MPa hydrogen pressure, localized cleavage surfaces begin to appear (Figure 11n), and at 10 MPa hydrogen pressure, the HAZ becomes entirely brittle (Figure 11o). According to the data presented in Table 6, the reduction in the area for the HAZ of the circumferential weld in a 10 MPa hydrogen environment is only 7.23%, significantly lower than 16.74% observed for the base material. This discrepancy suggests that the HAZ has a higher susceptibility to hydrogen embrittlement, potentially due to the hydrogen trapping effect at the coarse austenite grain boundaries within the HAZ (Figure 7).

**Figure 12:**
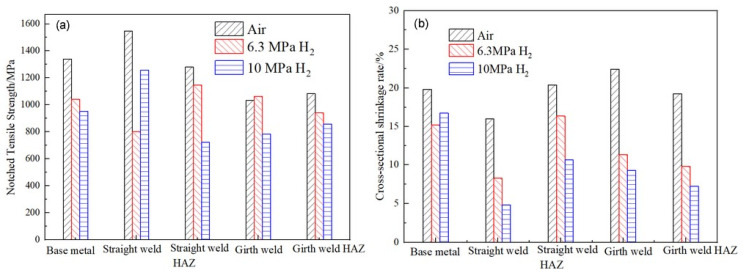
A performance comparison data of the X52 steel pipe at different positions under different hydrogen partial pressures: (**a**) notched tensile strength; (**b**) cross-sectional shrinkage rate.

The relevant mechanical properties of the material can be obtained through tensile curves and fracture measurements, as shown in Table 6. Figure 12 can be derived from the data presented in Table 6. As shown in Figure 12, compared with the air environment, the notch tensile strength and cross-sectional shrinkage of the material are significantly reduced under hydrogen pressures of 6.3 MPa and 10 MPa.

**Figure 13:**
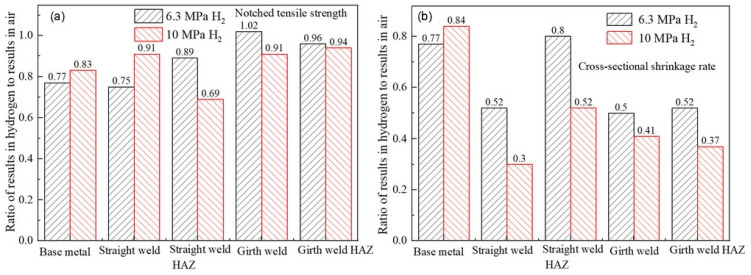
Performance comparison of steel pipes at different positions in hydrogen environment and air conditions: (**a**) notched tensile strength; (**b**) cross-sectional shrinkage rate.

According to the relevant standards and literature reviews [49,62], the hydrogen embrittlement sensitivity can be evaluated by the mechanical properties of identical specimens exposed to hydrogen-containing environments and non-hydrogen (ambient) environments. The greater the deviation of the ratio, the higher the cracking sensitivity. Based on the results in Table 6, the ratios of the notch tensile strength and reduction in the area between hydrogen-containing environments and air are calculated, as shown in Figure 13. As observed from the figures, the notch tensile strength of the X52 steel at the straight weld and HAZ shows a significant decrease in hydrogen environments. The reduction in the area at the straight weld, girth weld, and girth weld HAZ is also significantly lower in both environments. The ratios of these indicators are all less than 1. Under 6.3 MPa hydrogen pressure, the reduction in the area ratios for the base metal, straight weld, straight weld HAZ, girth weld, and girth weld HAZ are 0.77, 0.51, 0.80, 0.50, and 0.52, respectively. Under 10 MPa hydrogen pressure, the reduction in the area ratios for these positions are 0.84, 0.30, 0.52, 0.41, and 0.37, respectively. The results indicate that the X52 steel pipe exhibits a higher hydrogen embrittlement sensitivity and a higher risk of hydrogen embrittlement under the 10 MPa hydrogen pressure environment.

### 4.5. Smooth Round-Bar Tensile Test

**Figure 14:**
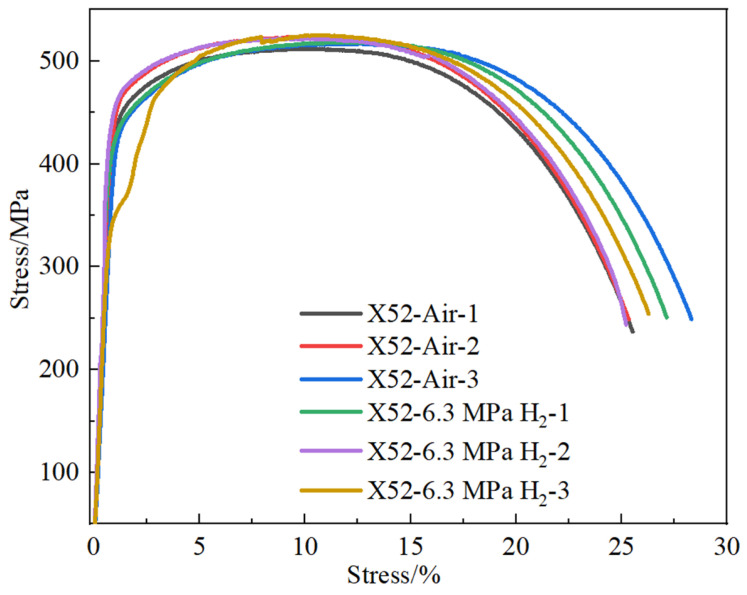
The tensile curve of the X52 steel pipe base metal smooth specimen in air and 6.3 MPa hydrogen environments.

**Figure 15:**
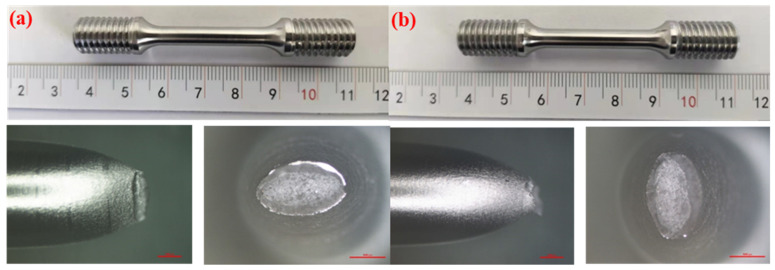
Macroscopic fracture morphology of the X52 steel pipe base metal smooth specimens in air and a 6.3 MPa hydrogen environments: (**a**) air; (**b**) 6.3 MPa H_2_.

The results of the notch tensile tests are relatively conservative, as the stress concentration at the notch location exhibits higher sensitivity to hydrogen. To accurately investigate the sensitivity of the X52 steel pipe in hydrogen environments, smooth round-bar tensile tests were performed on the base metal of the X52 steel pipe under 6.3 MPa hydrogen pressure. The tensile curves are shown in Figure 14. As seen from the figure, the tensile strength of the smooth tensile specimen of the X52 steel base metal fluctuates slightly in both air and 6.3 MPa hydrogen environments, indicating that the impact of 6.3 MPa hydrogen on the plasticity of the base metal is minimal, and the strain variation is also small. Macro-appearance observations of the tensile-tested smooth specimens are shown in Figure 15, where no brittle fracture zone was observed under hydrogen exposure.

**Figure 16:**
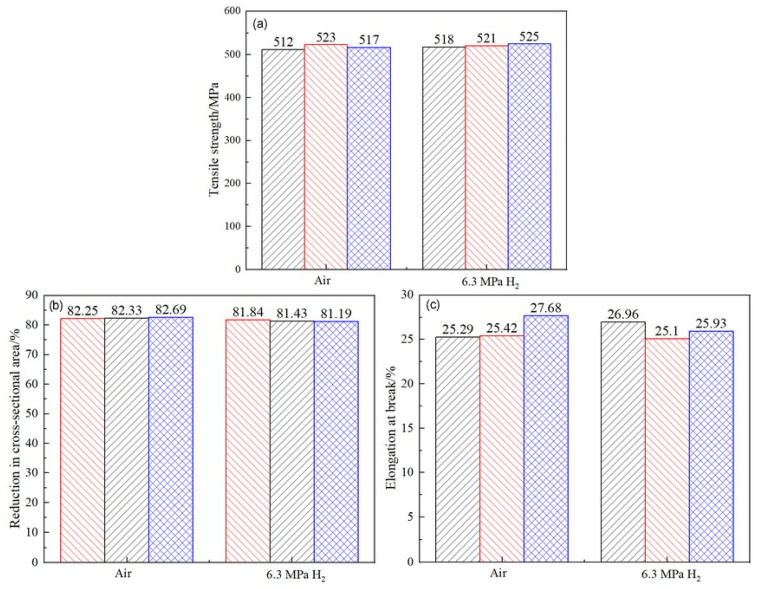
Comparative performance of the X52 steel pipe base metal in different environments. (**a**) Tensile strength; (**b**) reduction in area; (**c**) elongation after fracture.

By measuring the tensile curve and fracture, the related mechanical properties of the material can be obtained, as detailed in Table 7. It can be seen from Table 7 that the post-fracture elongation of the smooth round-bar specimens in air and hydrogen environments is not much different. Although the post-fracture elongation in the hydrogen environment is slightly higher than that in the air environment, the reason is that the different sampling positions of the materials will lead to differences in the internal microstructure of the specimens, which further affects the tensile properties of the materials. Coupled with the testing error of the instrument, the above-mentioned abnormal result eventually occurred. It can also be seen from Figure 14 that hydrogen has a weak influence on the post-fracture elongation of the material. Comparisons of the mechanical performance data are shown in Figure 15. Figure 16 indicate that the tensile strength, reduction in area, and elongation after a fracture of the smooth specimen of the X52 base metal show minimal changes.

**Figure 17:**
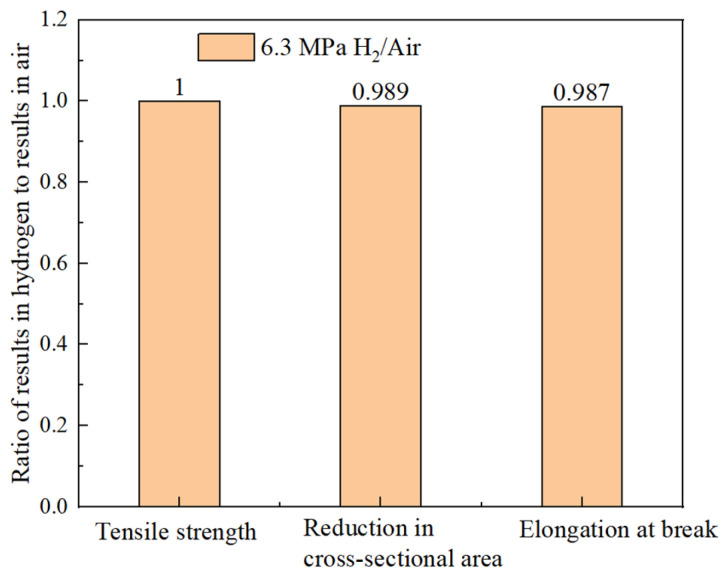
The ratio of different mechanical performance indices of X52 base metal smooth specimens in 6.3 MPa hydrogen environment and air environment.

The hydrogen embrittlement sensitivity can be evaluated by the mechanical properties of identical specimens exposed to hydrogen-containing environments and non-hydrogen (ambient) environments. The greater the deviation in the ratio, the higher the cracking sensitivity. Based on the results in Table 7, the ratios of the tensile strength, reduction in area, and elongation after a fracture between the hydrogen-containing environments and air are calculated. As shown in Figure 17, the ratios for the different indicators in both environments are close to one, indicating that the hydrogen embrittlement risk for the smooth specimen of the X52 base metal in a 6.3 MPa hydrogen environment is relatively low.

**Figure 18:**
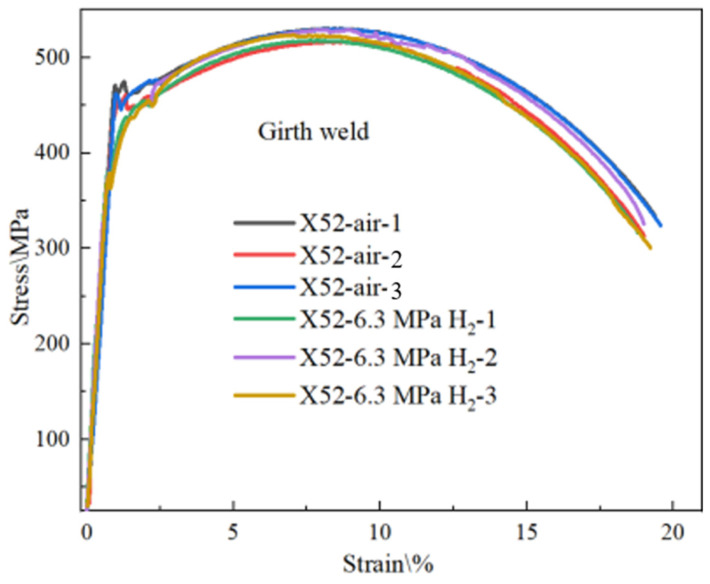
Tensile curves of pipe girth weld smooth specimens in air and 6.3 MPa H_2_.

**Figure 19:**
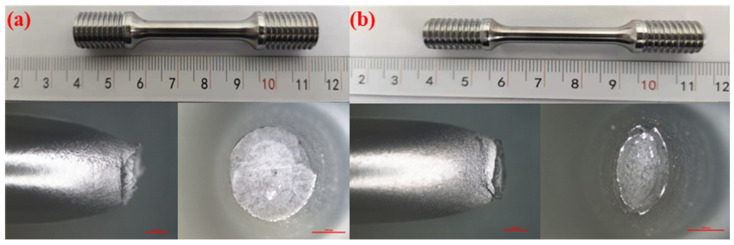
Macroscopic fracture morphology of X52 steel pipe girth weld smooth specimens in air and 6.3 MPa hydrogen environments: (**a**) air; (**b**) 6.3 MPa H_2_.

In order to accurately investigate the sensitivity of the X52 steel pipe’s girth weld in a hydrogen environment, smooth round-bar tensile tests were conducted on the X52 steel pipe’s girth weld under 6.3 MPa hydrogen conditions. The tensile curve is shown in Figure 18. As seen from the figure, the tensile strength of the smooth tensile specimens from the X52 steel pipe’s girth weld shows some fluctuation in both air and 6.3 MPa hydrogen environments, but the variation is small, indicating that 6.3 MPa hydrogen has little impact on the plasticity of the base metal smooth specimens. The strain values show minimal changes under the 6.3 MPa hydrogen environment. Macroscopic observations of the fractured smooth specimens are shown in Figure 19, where no brittle fracture zones were observed in the hydrogen environment. The relevant mechanical property indicators of the materials were obtained through tensile curve and fracture measurements, as shown in Table 8.

**Figure 20:**
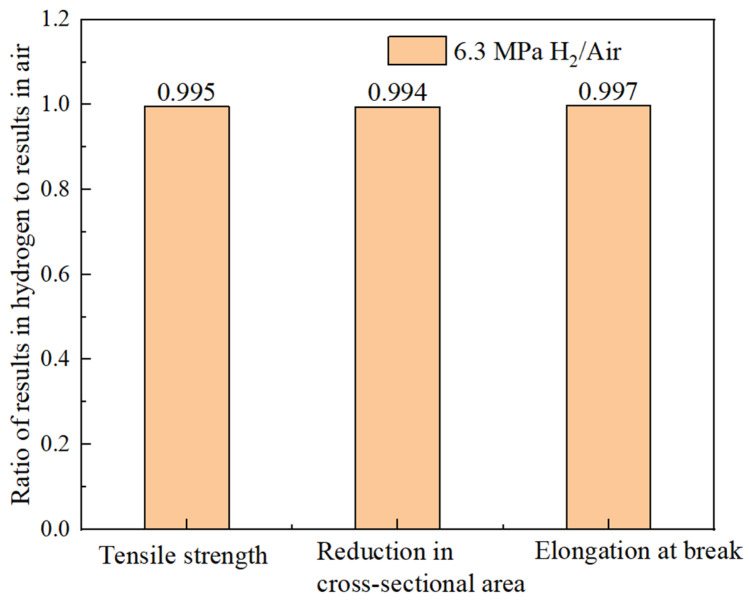
The ratio of different mechanical performance indices for X52 girth weld smooth specimens in 6.3 MPa hydrogen environment and air environments.

Hydrogen embrittlement sensitivity can be evaluated by comparing the mechanical properties of identical specimens exposed to hydrogen and non-hydrogen (normal temperature and pressure) environments. The ratios of the tensile strength, reduction in area, and elongation after a fracture in hydrogen environments to those in air are calculated and shown in Figure 20. It can be seen that the ratios of different indicators in both environments are close to one, indicating that the hydrogen embrittlement risk of the X52 steel base metal smooth specimens is relatively low under 6.3 MPa hydrogen partial pressure conditions.

### 4.6. Fracture Toughness Test

**Figure 21:**
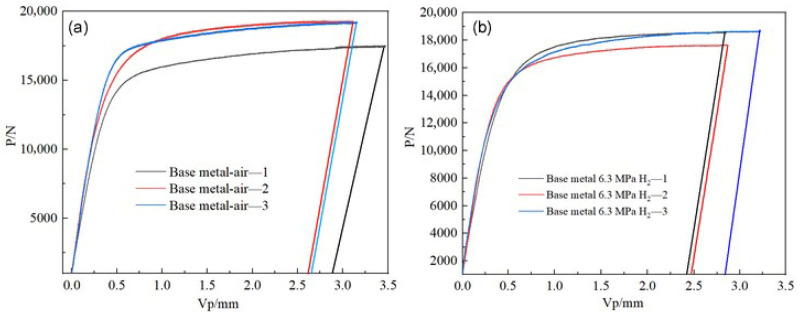
The P-V curves of the X52 steel base metal under different environments. (**a**) air; (**b**) 6.3 MPa H_2_.

The fracture toughness tests of the X52 steel base metal under both ambient conditions and 6.3 MPa hydrogen partial pressure were conducted using the CTOD (crack tip opening displacement) method. The F-V curves at different loading levels are shown in Figure 21. The corresponding force (F) and plastic displacement component (Vp), which is the intersection point of the F-V curve with the horizontal axis, were used to calculate the respective δ values for each sample’s shutdown point. The calculation results are presented in Table 9.


(1) $$\begin{array}{c} \delta ={\left[\frac{F}{{\left({BWB}_{N}\right)}^{0.5}}\times g2\left(\frac{a}{W}\right)\right]}^{2}\left[\frac{\left(1-\vartheta^{2}\right)}{{2ER}_{P0.2}}\right]+\frac{\left(R-a-Z\right)}{R}\; \end{array}$$

In the formula, μ is the Poisson’s ratio, taken as 0.3; σ_y_ is the yield stress, with the actual yield strength of X52 steel pipe taken as 455 MPa; *E* is the Young’s modulus, taken as 2.06 × 10^5^ MPa; *Z* is the notch thickness, taken as 0 mm; *W* is the specimen width, 24 mm; *a*_0_ is the initial crack length of the specimen, 12 mm.

**Table 9:** Loading data of fracture toughness tests for X52 steel base metal.

Material	Test Environment	*V_p_*/mm	*P*/N	*δ*/mm
X52 base metal	air—1	3.02	17,535	1.28
	air—2	2.7	19,321	1.16
	air—3	2.77	19,252	1.19
	6.3 MPa H_2_—1	2.39	18,601	1.03
	6.3 MPa H_2_—2	2.49	17,676	1.06
	6.3 MPa H_2_—3	2.82	18,671	1.20

The CTOD values for the two conditions are shown in Table 10. As the hydrogen content increases, the material’s *K_Q_* values remain relatively unchanged. According to the pipeline steel requirements for pipeline transport, the CTOD value must exceed 0.254 mm for the pipeline to be usable. All four conditions meet this requirement. The *K_Q_* values were calculated according to the relevant provisions in ASME B31.12, where *K_Q_* values were calculated using F_Q_ values. The results show that the CTOD values of the X52 steel base metal in air and 6.3 MPa hydrogen environments are 1.21 mm and 1.10 mm, respectively; the fracture toughness *K_Q_* values are 68.0 MPa·m^1/2^ and 66.6 MPa·m^1/2^, respectively. ASME B31.12 specifies that the *K_Q_* values are related to the sample thickness, with *K_IH_* values exceeding 55 MPa·m^1/2^. The *K_Q_* values for all four conditions fall within the standard range.

**Figure 22:**
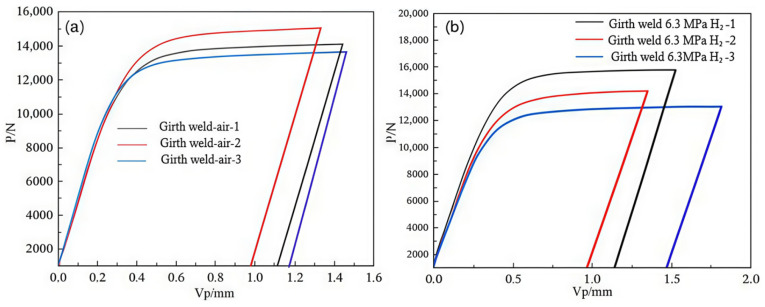
The P-V curves of the X52 steel girth weld under different environments: (**a**) air; (**b**) 6.3 MPa H_2_.

The fracture toughness tests of the X52 steel girth weld under H_2_-free and 6.3 MPa H_2_ environments were also performed using the CTOD method. The *F-V* curves at different loading levels are shown in Figure 22. Similarly, the force (*F*) and plastic displacement component (*VP*) at each sample’s shutdown point were used to calculate the respective δ values. The calculation results are shown in Table 11.

In the Formula (1), *μ* is the Poisson’s ratio, taken as 0.3; *σ_y_* is the yield stress, with the actual yield strength of the X52 steel pipe taken as 476 MPa; *E* is the Young’s modulus, taken as 2.06 × 10^5^ MPa; *Z* is the notch thickness, taken as 0 mm; *W* is the specimen width, 24 mm; *a*_0_ is the initial crack length of the specimen, 12 mm.

The CTOD values for the two conditions are presented in Table 12. As the hydrogen content increases, the material’s *K_Q_* values show little change. The CTOD values for all four conditions meet the usage requirements. According to ASME B31.12, the *K_Q_* values are calculated using *F_Q_* values. The results indicate that the CTOD values for the X52 steel girth weld in air and 6.3 MPa hydrogen environments are 0.47 mm and 0.51 mm, respectively, and the fracture toughness *K_Q_* values are 52.0 MPa·m^1/2^ and 52.3 MPa·m^1/2^, respectively. The ASME B31.12 standard states that *K_Q_* values are related to the sample thickness, and *K_IH_* values greater than 55 MPa·m^1/2^ are required. The *K_Q_* values for all four conditions are within the standard range.

The fracture toughness parameters of the X52 pipeline steel welds in hydrogen environments were measured and compared to those in air. The hydrogen environment was generated by on-site hydrogen charging, using a simulated soil solution as the electrolyte, with three different current densities: 1, 5, and 10 mA/cm^2^. A specially designed electrolytic cell mounted on a three-point bending setup was used to facilitate hydrogen charging during the monotonic loading of the samples. The measurement of ductility was based on the J0 integral. In all cases, slight changes in toughness were measured according to *K_Q_*. An increase in current density was associated with a reduction in ductility, particularly in the base metal. A more complex phenomenon was observed in the HAZ metal, where ductility slightly decreased at two current densities (1 and 5 mA/cm^2^), but the reduction was more pronounced at the third current density (10 mA/cm^2^). The hydrogen degradation effect was found to be more pronounced in the banded ferrite–pearlite microstructure, as observed through the microstructural analysis of the X52 base and HAZ metals.

The ferrite had a hardness of 154.5 HV, while the banded pearlite had a hardness of 245.5 HV. The average hardness of the X52 HAZ metal was 173 HV, with ferrite grains having a hardness of 160 HV and pearlite grains having a hardness of 212 HV. The HAZ exhibited a non-banded ferrite–pearlite microstructure, with grains of varying sizes [25].

**Figure 23:**
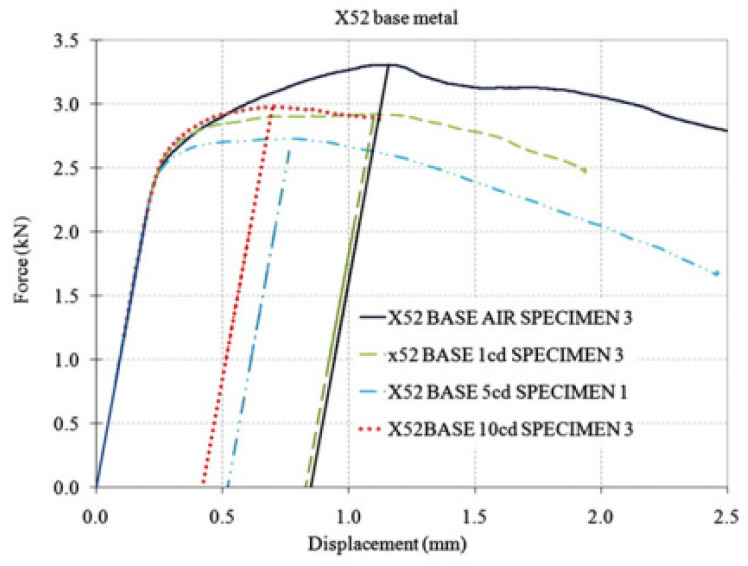
Load–displacement curves of X52 base metal in air and all hydrogen-charged conditions [25].

Fracture toughness tests were conducted on the X52 steel base metal and the HAZ under different hydrogen-charging concentrations. Figure 23 presents the force–displacement curves of the X52 base metal in air and under all hydrogen-charging conditions. The results of the X52 base metal samples are shown in Table 13. Regarding the base metal, the reduction in plasticity (as indicated by a decrease in *J*_0_) was seen with an increase in current density. Compared to *J*_0_ in air, the dispersion of the hydrogen-induced reduction in *J*_0_ at current densities of 1 and 5 mA/cm^2^ suggested a significant reduction in ductility (Table 14). At a current density of 10 mA/cm^2^, the average reduction in *J*_0_ was greater than that at 5 mA/cm^2^, but the dispersion of results can be attributed to the fact that, for a current density of 5 mA/cm^2^, critical hydrogen concentrations may have accumulated at the crack tip. Higher current densities facilitate greater hydrogen absorption, with minimal impact on fracture toughness.

**Figure 24:**
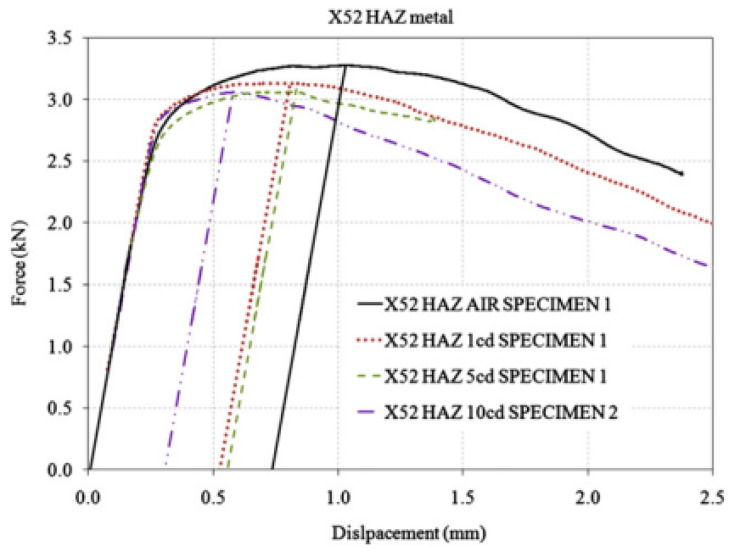
Load–displacement curves of the HAZ of the X52 steel pipe under air and all hydrogen-charged conditions [25].

Figure 24 shows the force–displacement curves for the X52 HAZ metal in air and under all hydrogen-charging conditions, and Table 15 presents the results of the X52 HAZ metal samples. Compared to the base metal, the HAZ demonstrated greater resistance to hydrogen-induced reduction in *J*_0_. As shown in Table 16, when a current density of 1 mA/cm^2^ was applied, the change rate in *J*_0_ was 6.4%, compared to 9.2% for the base metal. At a current density of 5 mA/cm^2^, the relative reduction in *J*_0_ for HAZ metal was 12.8%, while the base metal exhibited a relative reduction of 46.9%. Finally, for a current density of 10 mA/cm^2^, the relative reduction in *J*_0_ was more pronounced, at 33.4%.

The banded ferrite–pearlite microstructure of the X52 base metal affects hydrogen diffusion and permeation. During diffusion tests, it was found that hydrogen permeability was lower when diffusion occurred perpendicular to the bands compared to when it occurred parallel to the bands or in randomly distributed ferrite–pearlite microstructures, indicating that the pearlite layers act as barriers to hydrogen diffusion. The ferrite layers between the pearlite bands are described as preferential hydrogen diffusion paths. When hydrogen diffuses through the ferrite layers, the ferrite–pearlite interfaces become hydrogen-saturated, and hydrogen interacts with the deformation between the ferrite and pearlite bands.

**Figure 25:**
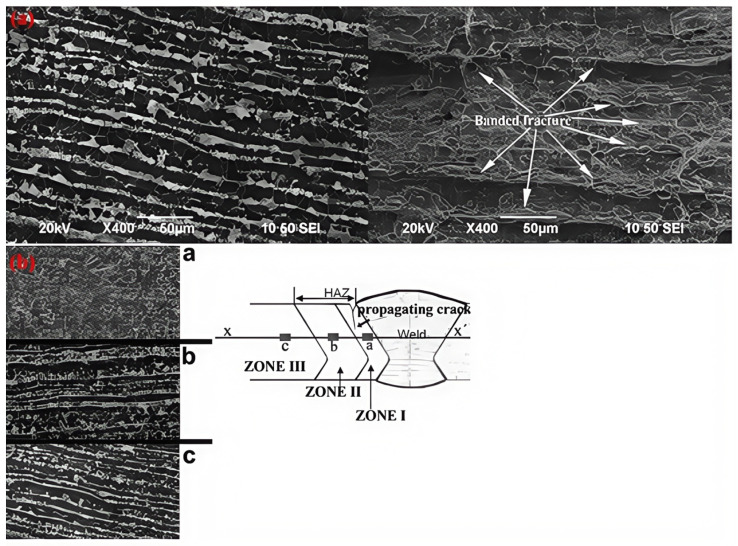
Microscopic images of crack propagation in the X52 steel pipe base metal and HAZ: (**a**) base metal; (**b**) HAZ [25].

Regarding the different behaviors of the X52 base metal and the X52 HAZ, there is a microhardness difference between the ferrite–pearlite bands in the X52 base metal and the ferrite–pearlite grains in the HAZ. The hardness difference between ferrite and pearlite in the X52 steel base metal is approximately 90 HV, while the hardness difference between ferrite and pearlite in the HAZ is about 52 HV. The values for the base metal suggest that there is a significant deformation gradient between the ferrite and pearlite bands, and the deformation gradient between the ferrite and pearlite grains in the HAZ appears to be enhanced by the hydrogen promotion effect at the ferrite–pearlite interfaces between the bands. The synergistic effect of hydrogen diffusion paths is increased by the banded structure and the higher ferrite–pearlite deformation gradient, which increases with hydrogen accumulation at the ferrite interface. To illustrate this, at a current density of 1 mA/cm^2^, the fracture morphology is characterized by cracks tearing into strips, perpendicular to the direction of the bag-shaped texture and parallel to the ferrite–pearlite banding, as shown in Figure 25.

The materials studied in the literature [25] are X52 steel pipes, where the base metal has a banded ferrite–pearlite microstructure. The HAZ has a non-banded ferrite–pearlite microstructure, with pearlite grains located further from the weld, and more pronounced banding effects in areas farther from the weld. This ferrite–pearlite banding causes the base metal to be more susceptible to hydrogen embrittlement than the HAZ metal. The continuous interfaces between ferrite and banded pearlite in the X52 base metal act as hydrogen traps, as hydrogen is forced to follow the ferrite bands during diffusion. With the aid of the large deformation gradient between the ferrite and pearlite bands, the interfaces become oversaturated, further increasing the deformation gradient. In contrast to the X52 base metal, the X52 HAZ exhibits a non-banded ferrite–pearlite microstructure, with smaller microhardness differences between the ferrite and pearlite grains, and greater resistance to hydrogen reduction in *J*_0_.

### 4.7. Fatigue Crack Growth Test

**Figure 26:**
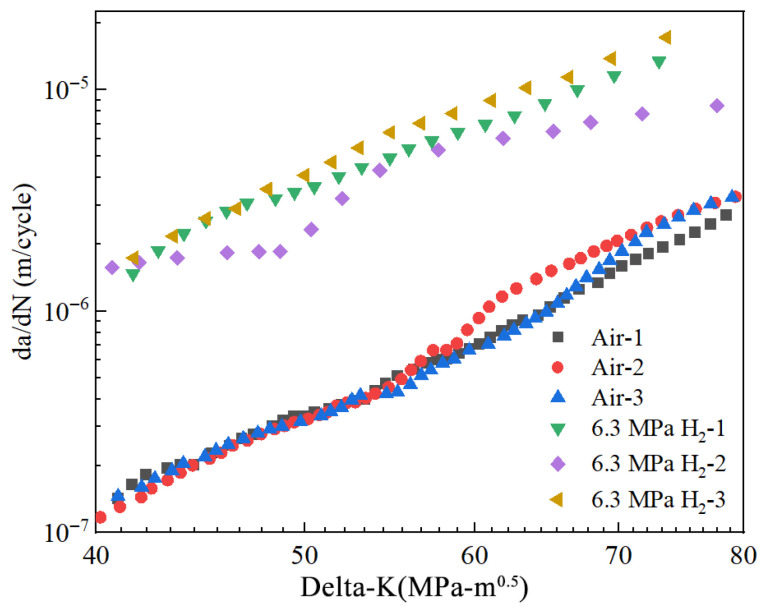
The fatigue crack growth curves of X52 steel pipe girth welds in hydrogen-free and 6.3 MPa H_2_ environment.

The fatigue crack growth curves of X52 steel pipe girth welds in a hydrogen-free environment and in a 6.3 MPa hydrogen environment are shown in Figure 26. The specific parameters used were a fatigue load stress ratio of 0.1 and a frequency of 1 Hz. As shown in the figures, the corresponding fatigue crack growth rates under 6.3 MPa hydrogen were 2.91 × 10^−6^ m/cycle and 1.44 × 10^−5^ m/cycle, representing an order of magnitude increase. The results indicate that hydrogen enhances the fatigue crack growth rate in X52 girth welds.

**Figure 27:**
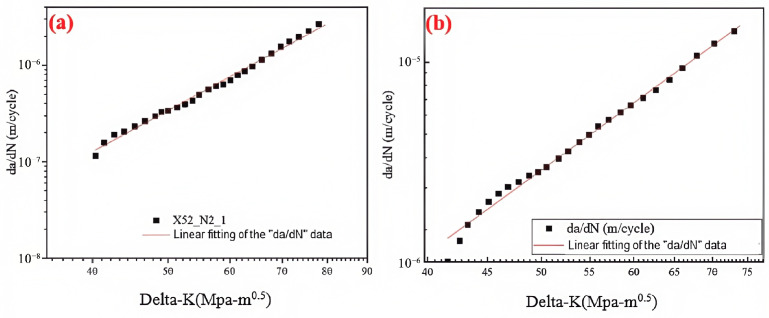
Fitted curve of the steel pipe girth weld in hydrogen-free and 6.3 MPa H_2_ environments: (**a**) hydrogen-free; (**b**) 6.3 MPa H_2_.

As the stress intensity factor range (Δ*K*) at the crack tip increases, the crack growth rate gradually increases at a constant rate. The relationship between the fatigue crack growth rate (*da/dN*) and the stress intensity factor (Δ*K*) was fitted using Paris’ law. Figure 27 shows the fitted curves under two different conditions. The parameters C and m were determined by the fitting equation, with values presented in Table 17. Based on the Paris’ law coefficients, it was found that the crack growth rate increases with the hydrogen partial pressure. Under the 6.3 MPa hydrogen condition, the fatigue crack growth rate was one order of magnitude higher than the rate observed in the absence of hydrogen.

## 5. Conclusions

This investigation identifies three key findings concerning the performance of X52 pipeline steel in hydrogen environments:

(1) Pressure-dependent embrittlement was clearly observed, with weld zones exhibiting significantly higher susceptibility compared to the base metal. At a hydrogen pressure of 6.3 MPa, cross-sectional shrinkage ratios remained comparable to those in air environments (ranging from 0.50 to 0.80 across all regions). However, at 10 MPa, welds showed severe degradation, with values as low as 0.30 for straight welds. This distinct pressure threshold effect should be incorporated into pipeline design standards.

(2) The base metal demonstrated excellent resistance to hydrogen-induced degradation, as evidenced by only minor differences in CTOD values (1.10 mm in hydrogen vs. 1.21 mm in air) and fracture toughness (66.6 vs. 68.0 MPa·m^1/2^). In contrast, girth welds exhibited a 4.95-fold increase in the fatigue crack growth rate under hydrogen exposure, indicating that current weld procedures may require optimization for hydrogen service applications.

(3) Based on fracture mechanics analysis, a critical CTOD threshold of 0.254 mm is proposed to ensure the structural integrity of X52 pipelines operating in hydrogen environments. This value incorporates safety margins derived from both base metal and weld region performance data.

(4) For the conversion of existing X52 pipelines to hydrogen service, reinforcement of weld regions or pressure derating may be necessary. In new pipeline designs, emphasis should be placed on preserving base metal properties and implementing more rigorous weld qualification protocols. The established 0.254 mm CTOD criterion serves as a quantitative basis for pipeline integrity management programs.

## Figures and Tables

**Table 1:** The chemical composition of X52 steel used in this study (%).

	C	Si	Mn	P	S	Cu	Ni	Cr	Mo	V	Nb	Ti	B	Pcm	Fe
X52	0.07	0.18	1.44	0.009	0.001	0.009	0.009	0.022	0.001	0.003	0.026	0.014	0.0001	0.15	98.0659

**Table 2:** The tensile properties of X52 steel used in this study.

	Yield Strength *R*_*t*0.5_ (MPa)	Tensile Strength *Rm* (MPa)	Elongation at Break *A* (%)
X52	433	555	46

**Table 4:** Mechanical property test results of X52 steel pipe base metal, longitudinal weld, and girth weld.

Position	Yield Strength (MPa)	Tensile Strength (MPa)	Elongation (%)
Base Metal	455	538	28.0
Straight Weld	453	537	22.5
Girth Weld	476	565	22.5
GB/T 9711 [61] Requirements	360–530	460–760	-

**Table 5:** Hardness values of X52 steel pipe longitudinal weld and girth weld at different sampling positions.

Vickers Hardness *HV*10							
N.O.		1	2	3	4	5	Ave.
Straight Weld	Weld Center	196.6	199.7	202.6	204.7	210.5	202.8
	HAZ	161.6	177.8	162.8	170.3	170.1	168.5
	Base Metal	165.1	165.3	162.6	165.9	165.1	164.8
Girth Weld	Weld Center	167.1	169.6	187.6	172.9	175.5	174.5
	HAZ	185.9	180.2	181.8	177.9	175.7	180.3
	Base Metal	182.5	187.1	178.9	183.9	176.1	181.7

**Table 6:** Notched tensile test results of X52 steel pipe base metal, longitudinal weld, heat-affected zone of longitudinal weld, girth weld, and heat-affected zone of girth weld under air and 10 MPa hydrogen gas conditions.

Position	Hydrogen Partial Pressure	Notch Tensile Strength	Cross-Sectional Shrinkage Rate
Base Metal	Air	1338 ± 0.05	19.81
	6.3 MPa H_2_	1041 ± 0.05	15.18
	10 MPa H_2_	1110 ± 0.05	16.74
Straight Weld	Air	1547 ± 0.05	15.98
	6.3 MPa H_2_	1160 ± 0.05	8.28
	10 MPa H_2_	1414 ± 0.05	4.83
HAZ of Straight Weld	Air	1280 ± 0.05	20.39
	6.3 MPa H_2_	1148 ± 0.05	16.37
	10 MPa H_2_	880 ± 0.05	10.65
Girth Weld	Air	1033 ± 0.05	22.45
	6.3 MPa H_2_	1063 ± 0.05	11.32
	10 MPa H_2_	940 ± 0.05	9.30
HAZ of Girth Weld	Air	1083 ± 0.05	19.29
	6.3 MPa H_2_	1039 ± 0.05	9.83
	10 MPa H_2_	1014 ± 0.05	7.23

**Table 7:** X52 mechanical property indices of the steel pipe base metal under different environments.

Test Environment	Tensile Strength	Reduction in Area/%	Elongation at Break/%
Air—1	512	82.25	25.29 ± 0.7
Air—2	523	82.33	25.42 ± 0.03
Air—3	517	82.69	27.68 ± 0.08
6.3 MPa H_2_—1	518	81.84	26.96 ± 0.06
6.3 MPa H_2_—2	521	81.43	25.10 ± 0.04
6.3 MPa H_2_—3	525	81.19	25.93 ± 0.03

**Table 8:** Mechanical property indices of the X52 steel pipe girth weld under different environments.

Test Environment	Tensile Strength	Reduction in Area/%	Elongation at Break/%
Air—1	530	72.55	19.21 ± 0.07
Air—2	516	72.42	18.66 ± 0.09
Air—3	530	72.35	18.90 ± 0.08
6.3 MPa H_2_—1	518	72.03	18.37 ± 0.08
6.3 MPa H_2_—2	528	71.86	18.43 ± 0.07
6.3 MPa H_2_—3	523	72.28	19.83 ± 0.09

**Table 17:** Paris’ law coefficients for crack propagation under different hydrogen gas environments.

H_2_ Atmosphere	*C*	*m*
H_2_ free	1.12 × 10^−14^	4.41
6.3 MPa	3.27 × 10^−12^	3.55

**Table 10:** Fracture toughness test characteristics of X52 steel base metal under different environments.

Test Environment	*δ_crit_*/mm	*K_Q_*
air	1.21	97.18
6.3 MPa H_2_	1.10	95.17

**Table 11:** Loading data of fracture toughness tests for X52 steel girth weld.

Material	Test Environment	*V_p_*/mm	*P*/N	*δ*/mm
X52 girth weld	air—1	1.10	14,145	0.47
	air—2	0.98	15,082	0.43
	air—3	1.17	13,693	0.50
	6.3 MPa H_2_—1	1.13	15,818	0.49
	6.3 MPa H_2_—2	0.96	14,225	0.42
	6.3 MPa H_2_—3	1.46	13,110	0.62

**Table 12:** Fracture toughness test characteristics of X52 steel girth weld under different environments.

Test Environment	*δ_crit_*/mm	*K_Q_*
air	0.47	74.34
6.3 MPa H_2_	0.49	74.74

**Table 13:** Fracture toughness parameter data of X52 base metal under different environments [55].

Specimens	$K_{Q}$ $\left(MPa\sqrt{m}\right)$	$J_{O}$ (N/mm)	$a_{O}$ (mm)	$a_{O}$ (W)	$F_{Q}$ (kN)	$F_{max}$ (kN)
Air						
1	45.2	151.9	6.8	0.6	2.0	2.5
2	44.9	148.1	5.8	0.6	2.8	3.7
3	45.1	141.8	6.2	0.6	2.4	3.3
1 mA/cm^2^						
1	46.1	126.2	6.3	0.6	2.4	2.9
2	46.3	138.0	6.7	0.6	2.1	2.6
3	46.1	137.0	6.2	0.6	2.5	3.1
5 mA/cm^2^						
1	45.8	83.6	6.3	0.6	2.4	2.7
2	45.0	69.2	5.8	0.5	2.9	3.2
3	45.4	81.8	6.6	0.6	2.1	2.5
10 mA/cm^2^						
1	43.3	73.7	6.0	0.6	2.5	3.1
2	41.7	71.0	5.8	0.6	2.6	3.2
3	45.7	71.7	6.3	0.6	2.5	3.0

**Table 14:** Variation in fracture toughness parameters of X52 base metal under different environments [25].

X52 Base Metal	$K_{Q}$ $\left(MPa\sqrt{m}\right)$	$J_{O}$ (N/mm)	$K_{Q}$ (%)	$J_{O}$ (%)
Air	45.1	147.3		
1 mA/cm^2^	46.1	133.8	+2.4	−9.2
5 mA/cm^2^	45.4	78.2	+0.8	46.9
10 mA/cm^2^	43.6	72.1	−3.3	51.1

**Table 15:** Fracture toughness parameter data of the HAZ of the X52 steel pipe under different environments [25].

Specimens	$K_{Q}$ $\left(MPa\sqrt{m}\right)$	$J_{O}$ (N/mm)	$a_{O}$ (mm)	$a_{O}$ (W)	$F_{Q}$ (kN)	$F_{max}$ (kN)
Air						
1	46.1	108.3	6.0	0.6	2.7	3.3
2	45.8	104.4	6.3	0.6	2.4	3.0
3	45.3	94.2	6.5	0.6	2.2	2.8
1 mA/cm^2^						
1	48.5	84.5	6.0	0.6	2.8	3.1
2	47.7	95.1	5.8	0.6	3.0	3.3
3	47.6	107.5	6.5	0.6	2.3	2.7
5 mA/cm^2^						
1	45.9	83.6	6.0	0.6	2.7	3.1
2	48.8	92.9	6.2	0.5	2.7	2.9
3	46.5	91.2	5.8	0.6	2.9	3.3
10 mA/cm^2^						
1	48.7	75.1	6.3	0.6	2.6	2.8
2	48.8	56.4	6.0	0.6	2.9	3.1
3	48.1	72.9	5.8	0.6	3.0	3.2

**Table 16:** Variation in the fracture toughness parameters of the HAZ of the X52 steel pipe under different environments [25].

X52 HAZ Metal	$K_{Q}$ $\left(MPa\sqrt{m}\right)$	$J_{O}$ (N/mm)	$K_{Q}$ (%)	$J_{O}$
Air	45.8	102.3		
1 mA	47.9	95.7	+4.7	−6.4
5 mA	47.1	89.2	+2.9	−12.8
10 mA	48.5	68.1	+6.1	−33.4

## Data Availability

The original contributions presented in this study are included in the article. Further inquiries can be directed to the corresponding authors.
